# Decision-making process about prenatal genetic screening: how deeply do moms-to-be want to know from Non-Invasive Prenatal Testing?

**DOI:** 10.1186/s12884-022-05272-z

**Published:** 2023-01-18

**Authors:** Serena Oliveri, Giulia Ongaro, Ilaria Cutica, Giulia Menicucci, Debora Belperio, Francesca Spinella, Gabriella Pravettoni

**Affiliations:** 1 Applied Research Division for Cognitive and Psychological Science, IEO, European Institute of Oncology IRCCS, Milan, Italy; 2 Department of Oncology and Hemato-Oncology, University of Milan, Milan, Italy; 3 Eurofins Genoma Group, Molecular Genetics Laboratories, Rome, Italy

**Keywords:** Prenatal testing, Decision making process, COVID-19, Prenatal genetic screening, Non-invasive prenatal testing

## Abstract

**Background:**

Prenatal information may be obtained through invasive diagnostic procedures and non-invasive screening procedures. Several psychological factors are involved in the decision to undergo a non-invasive prenatal testing (NIPT) but little is known about the decision-making strategies involved in choosing a specific level of in-depth NIPT, considering the increased availability and complexity of NIPT options. The main aim of this work is to assess the impact of psychological factors (anxiety about pregnancy, perception of risk in pregnancy, intolerance to uncertainty), and COVID-19 pandemic on the type of NIPT chosen, in terms of the number of conditions that are tested.

**Methods:**

A self-administered survey evaluated the decision-making process about NIPT. The final sample comprised 191 women (M_age_ = 35.53; SD = 4.79) who underwent a NIPT from one private Italian genetic company. Based on the test date, the sample of women was divided between “NIPT before COVID-19” and “NIPT during COVID-19”.

**Results:**

Almost all of the participants reported being aware of the existence of different types of NIPT and more than half reported having been informed by their gynecologist. Results showed no significant association between the period in which women underwent NIPT (before COVID-19 or during COVID-19) and the preferences for more expanded screening panel. Furthermore, regarding psychological variables, results showed a significant difference between perceived risk for the fetus based on the NIPT type groups, revealing that pregnant women who underwent the more expanded panel had a significantly higher level of perceived risk for the fetus than that reported by pregnant women who underwent the basic one. There was no statistically significant difference between the other psychological variables and NIPT type.

**Conclusions:**

Our findings indicate the paramount role of gynecologist and other health care providers, such as geneticists and psychologists, is to support decision-making process in NIPT, in order to overcome people’s deficits in genetic knowledge, promote awareness about their preferences, and control anxiety related to the unborn child. Decision-support strategies are critical during the onset of prenatal care, according to the advances in prenatal genomics and to parent’s needs.

**Supplementary Information:**

The online version contains supplementary material available at 10.1186/s12884-022-05272-z.

## Introduction

The number of *de novo* and inherited disorders and risk factors that can be detected through prenatal genetic testing is increasing rapidly, and in parallel, there is a growing desire to seek information about the physical health of the fetus [1], associated with the quick spread and massive increase in screening options [2, 3].

During the last decade, several studies have been performed on women’s decisions to undergo prenatal testing. According to the literature, three dimensions seem to predict the intention to undergo a prenatal genetic test: the need for more information about the fetus’ health, the positive attitude towards genetic tests, and the personal inclination towards the possibility of terminating the pregnancy after receiving a positive test result [4].

Several procedures are available to obtain prenatal information: invasive diagnostic procedures (such as chorionic villus sampling (CVS) and amniocentesis, both associated with iatrogenic pregnancy loss and miscarriage risk that is operator dependent approximately 1:200 [5]), and non-invasive screening procedures (such as those that use cell-free fetal DNA obtained from circulating maternal blood).

A recent systematic review [6] showed that the psychological factors related to the decision to undergo a prenatal test, both invasive and non-invasive, are: a need to have as much information as possible about the fetus health and a low tolerance to uncertainty. Several studies confirmed that the desire to reduce uncertainty about the health status of the fetus is positively associated with the decision of undergoing a prenatal genetic screening [6–11].

Low tolerance to uncertainty is the tendency to react “negatively to an uncertain event or situation, independent of its probability of occurrence and of its associated consequences” [12]. It is related with an individual’s need of being certain about their own capacity to cope with unpredictable change, and with adequate functioning in ambiguous situations. Another psychological factor related to the decision to undergo prenatal genetic testing is the anxiety level, defined as the tendency to experience anxiety-related thoughts and emotions in response to events involving risk or uncertain outcomes [13, 14]. It has been shown that undergoing prenatal testing may protect women from high levels of anxiety [15], or it might encourage them to focus on what may be wrong with the child, thus increasing levels of anxiety [16], at least until a reassuring result is received [17, 18]. Notwithstanding such juxtapositions in the impact of prenatal testing and anxiety relief or exacerbation, anxiety level during pregnancy is a factor that is important to be monitored, as high levels of prenatal anxiety and stress might have negative long-term consequences for both the pregnant woman and her fetus [19–21].

Within this framework, some studies specifically investigated the decision to perform invasive versus NIPT. Results show that the most important factors for preferring NIPT are: its high sensitivity, the fact that it could be performed at an earlier gestational age with respect to invasive tests, the absence of physical risks for the fetus, and the easiness of the procedure [6].The safety and non-invasiveness of NIPT, which are seen as a great advantage by pregnant women [22] likely cause less anxiety than an invasive test would.

To our knowledge, no studies have so far investigated the psychological variables that could affect the decision to perform a specific type of NIPT. Indeed, the different types of NIPT on the market offer several levels of investigation: common aneuploidies, rare aneuploidies, select structural chromosome anomalies, and partial deletions and duplications across all autosomes [3].

The main aim of this work is to assess, through the administration of standardized scales, several psychological variables, including anxiety about pregnancy, perception of risk in pregnancy, and intolerance to uncertainty, which might influence the choice of the type of NIPT in terms of the number of conditions that are tested. Indeed, we hypothesize that a higher need to have as much information as possible about the fetus health, a higher anxiety level, and a higher perception of pregnancy-related risks might increase the preference for panels testing for more conditions.

A further aim of this work is to explore the impact of the current health emergency linked to COVID-19 pandemic on the levels of risk perception in pregnancy and on the type of NIPT chosen. Indeed, it is known that the COVID-19 pandemic has resulted in the consolidation of a high degree of uncertainty worldwide [23–25], which also impacted on health behaviors [26, 27]. Furthermore, the uncertainty about the many unanswered questions regarding the impact of COVID-19 in pregnancy might have a role in the perception of vulnerability by pregnant women, therefore influencing their decisions regarding prenatal screening. Indeed, despite the growing number of published studies on COVID-19 in pregnancy, data do not allow to draw conclusions about the severity of the disease, the specific complications of COVID-19 in pregnancy, nor the vertical transmission [28, 29]. Notwithstanding, some studies suggest an increase in fetal, perinatal and neonatal complications such as abortion, preterm delivery, stillbirth, intrauterine growth retardation and fetal structural anomalies [30–33]. Furthermore, several studies revealed that pregnant women's anxiety level increased during the pandemic [34, 35]. It is thus possible that the higher uncertainty and the higher anxiety level reflects on the genetic conditions women decide to investigate through different types of NIPT.

## Material and methods

### Procedure

The current study was approved by the local Ethics Committee of the University of Milan (UNIMI- approval number 116/20).

For the study, female clients aged 18 years or older who underwent NIPT between November 2019 and May 2020 through Eurofins Genoma Group, a private genetic laboratory located in Rome and Milan, and had already completed their pregnancy were contacted by email and invited to participate in the study. Based on the test date, the sample of women was divided between “NIPT before COVID-19 group” (November 2019-February 2020) and “NIPT during COVID-19 group” (March 2020-May 2020), considering the Italian pandemic situation [25].

The main types of prenatal screening and related services provided by Eurofins Genoma Group laboratory to their customers, corresponding to different levels of detail, are as follows:

- Prenatalsafe: focuses on identifying common fetal chromosomal aneuploidies and severe genetic disorders in the fetus (trisomy 21,18,13,9,16), sex chromosomes aneuploidies and other six common microdeletion syndromes;
- Prenatalsafe Karyo: it screens for aneuploidies and structural chromosomal aberrations (deletion or duplications) across the fetal genome, it also analyzes 9 clinically significant microdeletion regions, providing information about gains or losses of chromosome material > 7 Mb across the fetal genome. It detects structural chromosome alterations at a resolution of approximately 3 Mb at the level of the chromosomal regions associated with the microdeletion syndromes investigated;
- Prenatalsafe Complete: it detects both genome-wide chromosomal abnormalities and single-gene disorders. It allows also detection of common inherited genetic disorders in the fetus that could be missed by traditional prenatal screening, such as Cystic Fibrosis, deafness autosomal recessive type 1A, deafness autosomal recessive type 1B, Thalassemia-Beta, Sickle cell Anemia, and de novo genetic conditions (e.g. cardiac defects, multiple congenital anomalies, and intellectual disabilities). It also analyzes 9 clinically significant microdeletion syndromes.

All female clients that were contacted had already provided, at the time of prenatal test, their consent to be re-contacted by the laboratory for research purposes. However, for this study, as per standard procedure, the research purposes and the procedure were explained through an information sheet and by a referred researcher and, if women agreed to participate in the study, they completed the informed consent form and subsequently received a link by email to fill in the online questionnaires. The questionnaires have been implemented on Qualtrics^TM^ Platform. Participants’ data were pseudo-anonymized and data collection was performed through an ID code (i.e., a combination of letters and numbers). Overall recruitment lasted from March 2021 to August 2021 (about a year later after they completed their prenatal screening). No compensation was provided to research participants for their engagement in the study.

### Participants

Two hundred one respondents gave their consent to participate but only 191 completed the questionnaire. Thus, the final sample of this study comprised 191 participants that were included in the analysis. Detailed socio-demographic characteristics of the participants are described in Table 1. All the participants were females, ranging from 25 to 55 years old (M_age_ = 35.53; SD = 4.79), and had already completed their pregnancy at the time of the enrollment. Specifically, nearly all of the sample (98.4%) carried the pregnancy to term; only 1.6% of pregnancies resulted in spontaneous abortion (SA) or therapeutic abortion (TA). The majority of respondents underwent NIPT for the first time (80.6%; *N* = 154) and 44% had previous pregnancies, ranging from 2 to 5 (M = 2.35; SD = 0.68). Previous pregnancies in only 13% of the sample resulted in SA or TA. Regarding the period in which they were tested, 61.3% of the participants (*N* = 117) underwent NIPT from March 2020 to May 2020, when COVID-19 pandemic had already hit Italian territory, whereas 38.7% (*N* = 74) of the participants underwent NIPT before COVID-19 pandemic (from November 2019 to January 2020). None of the women who underwent NIPT during COVID-19 pandemic tested positive or had close family members who tested positive. Almost all participants were in a stable relationship (married or cohabiting), were well-educated, and a high percentage of the respondents resided in Central Italy (46.6%; *N* = 89).

**Table 1:** Socio-demographic characteristics of the study sample

		Total Sample (*N* = 191)	NIPT before COVID-19 (*N* = 74)	NIPT during COVID-19 (*N* = 117)
Age	M ± SD, range	35.53 ± 4.79	35.69 ± 4.13	35.45 ± 5.18
		25–55		
Educational level				
	Primary/middle school	3.7% (7)		
	High school	30.4% (58)		
	Bachelor/Master’s Degree	50.3% (96)		
	Post PhD	15.7% (30)		
Marital Status				
	Single	6.8% (13)		
	Cohabiting/Married	92.1% (176)		
	Separated/Divorced	1.1% (2)		
Origin				
	Northern Italy	28.8% (57)	27% (20)	31.6% (37)
	Central Italy	46.6% (89)	52.7% (39)	42.7% (50)
	Southern Italy	23.6% (45)	20.3% (15)	25.7% (30)
Number of NIPT				
	First	42.4% (75)	77% (57)	82.9% (97)
	More than one	57.6% (102)	23% (17)	17.1% (20)
Type of NIPT				
	Pranatal Safe	42.5% (74)	35.7% (25)	47.1% (49)
	Prenatalsafe Karyo	48.9% (85)	60% (42)	41.3% (43)
	Prenatalsafe Complete	8.6% (15)	4.3% (3)	11.5% (12)

### Measures

The survey administered for this study was composed as follows:

- *Socio-demographic questions*: self-reported age, education, area of residence and marital status (4 questions) were assessed;
- *Previous pregnancies* and *experiences with NIPT*: Ten ad hoc items were created to assess a) primiparous or multiparous status; b) current pregnancies outcomes (“delivery” or “abortion”); c) previous pregnancies outcomes (“delivery” or “abortion”); d) previous NIPT experiences; e) type of NIPT panels performed (“Prenatalsafe”, “Prenatalsafe Karyo” or “Prenatalsafe Complete”); f) level of knowledge about NIPT panels (“Did you know the existence of different type of NIPT?”); g) source of information (“physicians”, “gynecologist”, “relatives and acquaintances” or “internet”); h) motivations for choosing that specific NIPT panel (“What led you to choose this type of NIPT specifically?”, answer options “The other panels were too expensive”, “The other panels were too detailed”, “The other panels were too poorly detailed”, “Further investigations would not have affected the choices related to pregnancy” and “I was mainly oriented by my gynecologist on the choice of this specific test”); i) motivation for deciding to undergo NIPT (an open-ended question coded into “advanced maternal age (1)”, “Additional assessment after previous instrumental investigation (2)”, “avoiding invasive diagnostic procedure (3)”, “need for information and increased awareness (4)”, “calming anxiety and reducing uncertainty (5), “family history or previous identified malformations/abortions (6)”); j) the level of involvement of the referred gynecologist (5 points Likert scale, from 1 “not at all involved” to 5 “totally involved”);
- *Social norms questions*: 4 questions investigated if the choice to undergo a NIPT was also related to the familiarity with this behavior, the influence of family members or significant others, and how much “socially” acceptable and appropriate the choice was perceived (1 multiple choice question, 1 Visual Analogue Scale (VAS), 2 items on a 5 point Likert scale from “completely disagree” to “completely agree”);
- *Baby subscale of the Anxiety Scale for Pregnancy* (ASP, [36]). The ASP is a measure of anxiety that covers multidimensional components of pregnancy and it is composed of 14 items 7 positively worded and 7 negatively worded, with items responses ranging from 1 “not at all” to 4 “very much”. The instrument has five subscales, covering the following dimensions of pregnancy: the baby (items 1, 6, 12), labor (items 2, 5, 14), marital (items 8, 10, 11), attractive (items 3, 13), and support (items 4, 7, 9). For this study, we only used the 3 items of the “baby” subscale, with the anxiety related to the unborn child. The subscale revealed a Cronbach’s alpha coefficient equal to 0.645 (rs > 0.361).
- *Perception of Pregnancy Risk Questionnaire* (PPRQ, [37]). The PPRQ is a self-report questionnaire consisting of 9 Visual Analogue Scales from 0 (no risk) to 100 (extremely high risk) to measure a pregnant woman's perception of her pregnancy risk, both for the child and herself. The PPRQ includes 2 dimensions: perceived risk to herself, 4 items, and perceived risk for the baby, 5 items. A total score had been calculated as a mean of the scores assigned to each item, with higher scores indicating higher perception of pregnancy risk. The total scale has been shown to be internally consistent (α = 0.862; rs > 0.434; *PPRQ Baby:* α = 0.874; rs > 0.591; *PPRQ Mother:* α = 0.642; rs > 0.364).
- *Intolerance of Uncertainty Scale-12* (IUS-12, [38, 39]). The IUS-12 is a short version of the original 27-item Intolerance of Uncertainty Scale [40, 41], and measures the intolerance to uncertainty through 12 items evaluated on a 5 point Likert scale from “completely disagree” to “completely agree”. Intolerance of uncertainty consists of a prospective factor (desire for predictability) and an inhibitory factor (uncertainty paralysis). The first dimension represents an active strategy for managing uncertainty and refers to the tendency to seek as much information as possible on situations perceived as threatening, in order to re-establish a condition of certainty. The second dimension, on the other hand, represents an avoidance strategy towards situations perceived as ambiguous, and it is converted into inability to act due to the uncertainty feelings. In this study, the total scale has been shown to be internally consistent (α = 0.888, rs > 0.319; *IUS Prospective:* α = 0.816; rs > 0.365; *IUS Inhibitory:* α = 0.901; rs > 0.687).

The group of women who underwent NIPT during COVID-19 pandemic (March 2020-May 2020) answered an additional set of 5 questions aiming at investigating their experience of worry related to the COVID-19 spread during their pregnancy. 2 items asked participants to evaluate from 0 to 10 (VAS) how much COVID-19 influenced their choice to undergo NIPT and how much COVID-19 might be a danger for pregnancy course. Furthermore, 3 items investigated about positivity to COVID-19 test of the participants, their partners or close family members, in the period preceding the NIPT. The response options for the 3 items will be binary coded (no—yes, specify).

### Data analysis

Data were analyzed using the statistical analysis software SPSS (Version 26.0, IBM, Armonk, NY, USA). Normality of the data was checked. Preliminary analyses including descriptive have been performed in order to characterize the participants. Chi-square tests for non-parametric factors, T-test analysis for independent groups and one-way analysis of variance (ANOVA) with Bonferroni correction have been performed to verify possible differences between groups in choices related to NIPT, motivations to undergo NIPT and in psychological variables such as anxiety, risk perception in pregnancy and intolerance to uncertainty.

## Results

### Impact of COVID-19 pandemic on NIPT decision-making process

A chi-square test was performed to examine the relation between COVID-19 and the type of NIPT chosen. Results showed that there was no significant association between the period in which women underwent NIPT (before vs during COVID-19) and the preferences for more expanded screening panel, even if participants who underwent Prenatalsafe Karyo were mainly pre-COVID-19 [*χ*^2^ (3, *N* = 177) = 8.722; *p* = 0.033)]. Nevertheless, COVID-19 pandemic did not impact on the decision to have more information about the genetic condition of the fetus. When questioned about the impact of COVID-19 on the decision to undergo NIPT, participants on average indicated no impact or very low impact (0.5 out of 10; SD = 1.08), despite rating the risks of COVID-19 associated with their pregnancy as moderately high (4.56 out of 10; SD = 3.41).

### Motivational factors associated with NIPT type chosen

A chi-square test was performed to test how the type of motivation leading to the choice of a specific type of NIPT was distributed in our sample. Contingency tables showed that the relation between motivation and type of NIPT was significant, [χ^2^ (10, *N* = 191) = 37.542; *p* < 0.001)], i.e., motivations differ significantly depending on the type of test chosen. Specifically, the analysis of standardized residual indicated that women who choose the more expanded screening panel (Prenatalsafe Complete) reported more frequently that they made this choice because other panels were too poorly detailed (Prenatalsafe Complete: 60% [*n* = 9] vs Prenatalsafe: 5.4% [*n* = 4] and Prenatalsafe Karyo: 11.8% [*n* = 10]; adjusted standardized residual = 5.6), and less frequently that they were guided in this choice by their gynecologist (Prenatalsafe Complete: 26.7% [*n* = 4] vs Prenatalsafe: 54.1% [*n* = 40] and Prenatalsafe Karyo: 52.9% [*n* = 45]; adjusted standardized residual =—2.0).

Another Chi-Square Test of Independence was performed to assess the relationship between the motivation to undergo NIPT in general and the choice of a specific type of NIPT. Results showed that there was not a significant relationship between the two variables, [χ^2^ (10, *N* = 191) = 8.831; *p* = 0.54)], i.e., the motivation with which pregnant women undergo prenatal testing did not differ depending on the type of NIPT chosen.

Furthermore, almost all of the participants (94.8%; *N* = 181) reported being aware of the existence of different types of NIPT that can provide information about a range of different genetic conditions, and more than half (72.3%, *N* = 138) reported having been informed by their gynecologist. Only a minority reported having sought information on their own through websites (16.2%; *N* = 31) or through friends and acquaintances (6.3%; *N* = 12). With respect to the degree of involvement of their gynecologist, more than half reported that their gynecologist suggestion was decisive in deciding which type of NIPT to choose. With respect to the role of their gynecologist in the decision-making process, more than half (53.4%; *N* = 102) reported that their gynecologist was very or totally involved in the choice of NIPT type (neither too much nor too little: 18.8%, *N* = 36; little/not at all: 27.8%, *N* = 53). However, although the contingency tables showed that the relation between the degree of involvement of the gynecologist and type of NIPT was not significant, [χ^2^ (4, *N* = 174) = 6.898; *p* = 0.141)], the analysis of standardized residual indicated that those who choose the Prenatalsafe Complete reported more frequently that they chose it without any involvement of their gynecologist in their decision (Prenatalsafe Complete: 53.3% [*n* = 8] vs Prenatalsafe: 28.4% [*n* = 21] and Prenatalsafe Karyo: 24.7% [*n* = 21]; adjusted standardized residual = 2.2).

### Psychological variables associated with NIPT type chosen

Psychological variables of the sample were summarized in Table 2. Results showed a significant difference between perceived risk to the fetus (PPRQ Baby) based on the NIPT type groups, as demonstrated by the one-way ANOVA (F_(2,167)_ = 4.22, *p* = 0.016).

**Table 2:** Means and standard deviations of the main psychological variables

**Psychological Variables** (*N* = 187)	**M**	**SD**
**IUS**		
IUS Total	35.32	8.31
IUS Prospective	23.43	5.02
IUS Inhibitory	11.89	4.32
**PPRQ**		
PPRQ Total	35.71	21.31
PPRQ Baby	43.58	28.11
PPRQ Mother	25.87	20.95
**ASP**		
ASP Baby	2.15	.67

Bonferroni corrected pairwise comparisons revealed that the pregnant women who underwent the expanded panel (Prenatalsafe Complete) had a significantly higher level of perceived risk to the fetus (M = 57.88; SD = 29.62) than that reported by the pregnant women who underwent the Prenatalsafe panel screening, the basic one (M = 37.77; SD = 26.17). There was no statistically significant difference between the other psychological variables and NIPT type (IUS Total: F_(2,164)_ = 0.57, *p* = 0.944; IUS Prospective: F_(2,164)_ = 0.27, *p* = 0.763; IUS Inhibitory: F_(2,164)_ = 0.35, *p* = 0.965; PPRQ Total: F_(2,167)_ = 3.58, *p* = 0.03; PPRQ Mother: F_(2,164)_ = 0.84, *p* = 0.433; ASP Baby: F_(2,164)_ = 2.83, *p* = 0.061).

Results showed a significant effect of NIPT type groups on levels of perceived social norms, F_(2,171)_ = 6.51, *p* = 0.002. Pairwise comparisons were conducted on all the pairs of groups using the Bonferroni correction. Post hoc tests revealed that participants in the Prenatalsafe Karyo group had significant less adherence to social norms (M = 2.91, SD = 1.08) than those in the Prenatalsafe group (M = 3.53, SD = 1.11), whereas participants in the Prenatalsafe Complete group (M = 2.93, SD = 1.98) did not significantly differ from the other two groups.

Furthermore, Chi-Square Test of Independence showed that there was not a significant relationship between past pregnancy outcomes, and the choice of a specific type of NIPT [χ^2^ (2, *N* = 77) = 2.16; *p* = 0.34)], as well as between being primiparous or multiparous and the choice of a specific type of NIPT [χ^2^ (2, *N* = 174) = 1.61; *p* = 0.44)]. As demonstrated by the one-way ANOVA, no association between age and the choice of a specific type of NIPT was found (F_(2,171)_ = 0.668, *p* = 0.514).

## Discussion

Until more recent times, the risks associated with the execution of invasive prenatal tests (amniocentesis or CVS) oriented health professionals to direct the prenatal diagnosis only to the female population selected as "at risk". Now NIPT exams are aimed at the whole population, since they are characterized by simplicity of execution and risk-free. Nevertheless, with increased complexity and availability of NIPT options [42], decision-support strategies are critical to promote informed decision making in women and elicit their preferences regarding the use of these screens. General population commonly struggle with understanding key informational genetic aspects and often lack the health literacy and numeracy skills to interpret and personalize the risk information [43–49], such as the basic distinction between aneuploidy screening (AS), that focuses on identifying chromosomal aneuploidy (e.g., trisomy 21) and other genetic abnormalities such as de novo mutations, microdeletions, or single gene disorders (SGD) that focuses on identifying heritable genetic mutations.

As we have seen from the literature, the choice to undergo a NIPT, like other prenatal tests, is mainly guided by the perception to maintain a greater control over pregnancy, reduce uncertainty and to make decisions about the future of pregnancy itself [6–11]. A recent study specifically investigated both patients' knowledge of prenatal genetic screening and their decision-making preferences for screening when offered an expanded screening panel [50]. In Farrell et al. [50] when expanded panels increased to contain 50 conditions, fewer participants preferred to learn about all of the conditions on the panel, while the most of participants expressed their preference to learn about conditions post-test, based on the results received. Instead, participants who at the beginning preferred to limit pre-test education to conditions at which they were at risk for did not change significantly their preference when offered panels increased e.g. from 5 to 100 conditions [50].

In our study we investigated more in-depth whether psychological factors, in particular the anxiety levels towards the fetus, perception of pregnancy risk related to the mother or the fetus, adherence to social norms and intolerance to uncertainty, affect the choice to undergo an expanded screening panel. The study was also conducted during a stressful event that affected the whole world, the COVID-19 pandemic. Almost the entire sample of participants in our study reported being aware of the existence of different types of NIPT that can provide information about a range of different genetic conditions, and the most had been informed by their gynecologist. However, the awareness of the existence of different screening panels cannot be confused or interpreted as a greater knowledge of the genetic conditions being examined and their implications. As demonstrated in the study by Farrell et al. [50], women are more familiar with the concepts associated with AS (e.g. risk of trisomy 21, 18, or 13) compared to CS (e.g. cystic fibrosis, sickle cell anemia or thalassemia). This lack of knowledge concerns several aspects, among which the interpretation of screening results and the implications of such risk assessments on their pregnancy and future reproductive decision-making [51–53].

The percentage of women who underwent an expanded screening panel in our study (Prenatalsafe Complete) was overall low (8.6%). Their main reported motivations for choosing Prenatalsafe Complete were that other screening panels were less detailed compared to the information they wanted to gather about risks for their child, as well as the lack of engagement of their gynecologist in the decision-making process. They also had a significantly higher perception of risk for the baby than those who choose Prenatalsafe or Prenatalsafe Karyo. Although there were few subjects to infer something, results showed that their choice was not guided by previous abortion experiences, by primiparity, or by advanced maternal age. In Farrell et al. [50] it was instead observed a difference between primiparous and multiparous, with the first having greater preferences to learn about more genetic conditions, in particular those that would lead to the death of a child soon after birth or severely affect the quality of life (QoL) of a child.

Our results suggest that the expanded genetic panel Prenatalsafe Complete is chosen without the specific guidance of medical indications and on the basis of emotional factors such as anxiety related to the unborn child. However, the presence and experience of the COVID-19 pandemic did not play any significant role in the emotional reactions and in the perception of higher genetic risk for the child, except for the concern related to pregnancy pathway and the opportunity to be adequately followed with regular medical visits.

Following good practice guidelines, women might consider to be tested with NIPT for conditions at which they were at risk for, based on what they would be willing to do with the test result, and well discussed with referred clinicians (gynecologist, genetic counselor, family physicians) [54]. In our study, indeed, the women who decided to undergo Prenatalsafe Karyo genetic screening were mainly guided by the indications of their gynecologist, compared to those who instead chose more expanded screening panels. Furthermore, they were women who, although not clearly adhering to social norms, appear to have a significantly greater tendency to follow social norms than those who undergo the Prenatalsafe Complete panel. This could suggest an effect of social norms and values ​​in limiting the freedom of women to decide for an expanded genetic panel, and therefore the freedom to receive more information in order to decide for a possible therapeutic abortion.

Concerning the first result, in other studies conducted on the Italian population the dominant role of the specialized physician emerged [55–57], also referred to the choice of undergoing genetic testing for personal disease risk calculation, and it was prevalent compared to other cultural contexts [58, 59].

As for adherence to social norms, the debate is much more complex. As reported by Stapleton [60] and van Schendel et al. [61] a prenatal test like NIPT cannot predict the disease severity or the QoL of the child, that is also a relatively subjective concept and differs per person, for this reason every “*Women should be able to make their own decision about what to test for and what not to test for*” [61]. Farrimond and Kelly [62] noted that for a minority of their participants “*it should be the parents’ decision what tests to have and what they want to do with the results* (P22, female, currently pregnant)” [62]. In this framework social norms and values should not conflict with the full range of screening options that could be offered.

Furthermore, based on a societal point of view, NIPT and decisions about selective abortion are a public health issue [63]. Some researchers argued that a woman who makes use of NIPT is seen as a responsible pregnant woman [64] who is acting in the best interests of the fetus, her family, and her community [65]. Conversely, a  pregnant woman who either does not comply with a referral for testing or decides to continue to carry a fetus in which a disability has been detected is viewed by others as irresponsible, irrational, and selfish [66]. These debates illustrate the importance of closely monitoring the prevailing social norms governing NIPT use, in terms of both societal opinion and the actual allocation of social resources as the technology advances. True reproductive autonomy necessarily involves striving for a social context in which parents who choose not to undergo testing, or who choose to raise a child with a disability, or on the contrary decide to undergo a therapeutic abortion, would be supported.

Our findings indicate the paramount role of gynecologist and other health care providers, such as geneticists and psychologists, to support decision making process in NIPT, in order to overcome people’s deficits in genetic knowledge, promote awareness about their preferences, control anxiety related to the unborn child. Further studies about parent’s preferences and psychological profiles, along with innovative approaches, are needed to best support parent’s informed decision-making about an expanding array of NIPT options at the onset of prenatal care.

The present study has some limitations and should be interpreted with caution. Data were collected using a self-administered survey among eligible women who gave their free consent to participate and this might have created a selection bias among the types of participants who completed the survey and the type of NIPT panel they underwent. Secondly, the small sample size did not allow any causal inference but only observational exploration. Furthermore, although Eurofins Genoma Group laboratory makes counselling available both pre-test and for high risk results, data on genetic counselling sections and its impact on the decision-making process were not recollected.

## Conclusion

We highlighted the importance of psychological aspects, such as high levels of anxiety related to the unborn child or the adherence to social norms, in determining a certain autonomy of choice for women/parents in the domain of and expanded array of prenatal genetic screening. Along with the importance of providing genetic education to parents about the different genetic condition (e.g. AS and SGD), such results and future researches have to foster healthcare providers and systems to revise how to structure the medical decision-making processes during the onset of prenatal care, according to the advances in prenatal genomics and to parent’s needs.

## Supplementary Information

**Additional file 1.**

## Data Availability

The datasets generated and/or analysed during the current study are not publicly available due to privacy concerns related to the presence of sensitive data but are available from the corresponding author on reasonable request.

## Abbreviations

AS: Aneuploidy Screening
ASP: Anxiety Scale for Pregnancy
CS: Carrier Screening
CVS: Chorionic Villus Sampling
IUS: Intolerance of Uncertainty Scale
NIPT: Non-Invasive Prenatal Testing
PPRQ: Perception of Pregnancy Risk Questionnaire
QoL: Quality of Life
SA: Spontaneous Abortion
SGD: Single Gene Disorders
TA: Therapeutic Abortion
VAS: Visual analogue Scale
