# Supplementary material for: Decision-making process about prenatal genetic screening: how deeply do moms-to-be want to know from Non-Invasive Prenatal Testing?
Source: BMC Pregnancy Childbirth. 2023 Jan 18;23:38. doi: 10.1186/s12884-022-05272-z (PMC9845820; doi:10.1186/s12884-022-05272-z)
Supplement: Supplementary file 1 — Additional file 1. [file 12884_2022_5272_MOESM1_ESM.pdf]

Supplementary Material

Attachment A: Questionnaires

Age ____

What is your highest level of education?

- o Primary/Middle School
- o High School
- o Bachelor/Master's Degree
- o Ph.D or Post Ph.D.

What region do you come from?___________________

On what date did you undergo your NIPT (month and year)? _______________________

What was your marital status at the time the test was taken?

- o Unmarried
- o Cohabiting
- o Married
- o Separated/divorced
- o Widowed

Have you performed any other prenatal genetic tests before this one?

- o Yes
- o No

Was the pregnancy for which you underwent a NIPT at GenomaLab your first pregnancy? If no, please specify (e.g., second)

- o Yes
- o No __________________

How did the previous pregnancy(s) end? Multiple answers can be selected

- o natural/cesarean delivery
- o Spontaneous/therapeutic abortion

The pregnancy for which you underwent a NIPT at GenomaLab, how did it end?

- o Natural/cesarean birth
- o Spontaneous abortion/therapeutic abortion

What kind of NIPT have you done?

- o Prenatal Safe
- o Prenatal Safe Karyo
- o Prenatal Safe Complete
- o Other: specify__________________________

Were you aware of the existence of different levels of in-depth NIPT?

- o Yes

o No

If yes, who told you about it?
o The family doctor
o The gynecologist
o Family members/friends/acquaintances
o I gathered information on my own through the internet

What led you to choose this type of NIPT specifically?
o The others (more in-depth) were too expensive
o The others were too detailed
o The others were too little detailed
o Further investigation would not have affected my pregnancy decisions
o I was mostly directed by my gynecologist on the choice of this specific test
o Other: specify ___________________________

How involved was your gynecologist in the decision-making process about the type of NIPT?
o Not at all
o A little
o Neither a little nor a lot
o Very much
o Totally

Why you decide to undergo a NIPT? ____________________________

Among the people who have gone through pregnancy that you know (friends, family members, acquaintances), how many have undergone prenatal genetic testing?
o All of them
o More than half
o About half
o Less than half
o None

We ask you to read the following statements and indicate, for each, your level of agreement.

	Completely disagree	Somewhat disagree	Neither agree nor disagree	Somewhat agree	Completely agree
All pregnant women undergo prenatal genetic testing					
People around me expected me to undergo prenatal genetic testing					
In my experience, undergoing prenatal genetic testing is "the norm"					

Considering your decision to undergo a NIPT, how much did the thoughts of people close to you (e.g., partners, family members, friends, colleagues) influence your decision? From 0 (not at all) to 10 (totally)

0 1 2 3 4 5 6 7 8 9 10

Anxiety Scale for Pregnancy (ASP)_ Subscale Baby

	Not at All	Somewhat	Moderately	Very Much
I feel relaxed about the health of my baby				
I feel nervous that my baby will have a deformity or a disease				
I feel confident that my baby will be born healthy				

Perception of Pregnancy Risk Questionnaire (PPRQ)

(1) The risk for myself during this pregnancy

0 10 20 30 40 50 60 70 80 90 100

(2) The risk for my unborn baby during this pregnancy

0 10 20 30 40 50 60 70 80 90 100

(3) The risk for my unborn baby during this pregnancy

0 10 20 30 40 50 60 70 80 90 100

(4) My risk of having a cesarean section

0 10 20 30 40 50 60 70 80 90 100

(5) My risk of dying during this pregnancy

0 10 20 30 40 50 60 70 80 90 100

(6) My baby's risk of being born prematurely

0 10 20 30 40 50 60 70 80 90 100

(7) My baby's risk of having a birth defect

0 10 20 30 40 50 60 70 80 90 100

(8) My baby's risk of needing to go to the Neonatal Intensive Care Unit

0 10 20 30 40 50 60 70 80 90 100

(9) My baby's risk of dying during this pregnancy

0 10 20 30 40 50 60 70 80 90 100

Intolerance of Uncertainty Scale (IUS-12)

	Not at all characteristic of me		Somewhat characteristic of me		Entirely Characteristic of me
1. Unforeseen events upset me greatly.	•	•	•	•	•
2. It frustrates me not having all the information I need.	•	•	•	•	•
3. One should always look ahead so as to avoid surprises.	•	•	•	•	•
4. A small, unforeseen event can spoil everything, even with the best of planning.	•	•	•	•	•
5. I always want to know what the future has in store for me.	•	•	•	•	•
6. I can’t stand being taken by surprise.	•	•	•	•	•
7. I should be able to organize everything in advance.	•	•	•	•	•
8. Uncertainty keeps me from living a full life.	•	•	•	•	•
9. When it's time to act, uncertainty paralyses me.	•	•	•	•	•
10. When I am uncertain I can’t function very well.	•	•	•	•	•
11. The smallest doubt can stop me from acting.	•	•	•	•	•
12. I must get away from all uncertain situations.	•	•	•	•	•

(only for pregnant women who underwent NIPT during COVID-19 pandemic)

How much from 1 (not at all) to 10 (totally) do you think COVID-19 influenced your choice to undergo a NIPT?

1 2 3 4 5 6 7 8 9 10

Before you underwent a NIPT, how much from 1 (not at all) to 10 (totally) did you think COVID-19 might pose risks to your pregnancy (e.g., safety in the hospital during screening, access to therapeutic treatments, etc.)?

1 2 3 4 5 6 7 8 9 10

Have you tested positive for COVID-19?

- Yes
- No

Have your partner tested positive for COVID-19?

- Yes
- No

Have any of your acquaintances tested positive for COVID-19?

- Yes
- No
